# Oral Immunotherapy Using Probiotic Ice Cream Containing Recombinant Food-Grade *Lactococcus lactis* Which Inhibited Allergic Responses in a BALB/c Mouse Model

**DOI:** 10.1155/2020/2635230

**Published:** 2020-09-24

**Authors:** Alireza Vasiee, Fereshteh Falah, Mojtaba Sankian, Farideh Tabatabaei-Yazdi, Seyed Ali Mortazavi

**Affiliations:** ^1^Department of Food Science and Technology, Faculty of Agriculture, Ferdowsi University of Mashhad, Mashhad, Iran; ^2^Immunology Research Center, Bu-Ali Research Institute, School of Medicine, University of Medical Sciences, Mashhad, Iran

## Abstract

This study was conducted to evaluate the effects of recombinant probiotic bacteria as a candidate for oral vaccine with the potential of treating allergy to *Amaranthus retroflexus* pollens. The main gene of this allergen, Ama r 2, was cloned into the food grade plasmid pNZ7025 and then was electrotransformed into the food grade *Lactococcus lactis* NZ1330. No expression was observed in the primary structure due to the distance between the ribosome binding site and the start codon. Therefore, the vector structure was corrected using the site-directed mutagenesis (SDM) technique. The cell extract of this strain was used for assessing the expression of the recombinant allergen in western blot analysis, and the existence of this protein with a molecular weight of 14.2 kDa was confirmed. To evaluate the efficacy of this strain in the treatment of allergies as an oral vaccine, probiotic ice cream was prepared. After the sensitization of mice, the treatment was performed by oral immunotherapy for 4 weeks, 4 to 5 times per week. 20 *μ*l of functional ice cream with 10^12^ CFU/ml of r-*L. lactis* NZ1330 significantly reduced the serum IgE level. The levels of IFN-*γ* and TGF-*β* cytokines increased in the 20 *μ*l ice cream treatment group as well as 40 *μ*g/ml pure allergen compared with the PBS-treated group, and IL-4 cytokine levels decreased compared with the PBS-treated group. Overall, 20 *μ*l ice cream with 10^12^ CFU/ml of the recombinant bacteria resulted in the best performance in terms of improving allergies to Th1 and Treg responses.

## 1. Introduction

Allergy is a disorder created when the immune system reacts to harmless antigens. Allergic diseases comprise heterogeneous inflammatory pathologies such as respiratory, cutaneous, and food allergy [1, 2]. 300 million and nearly 200-250 million people, respectively, suffer from asthma and food allergies all over the world. One tenth of the world population suffers from rhinitis and 400 million from drug allergies [3]. The allergy from the pollens of *Amaranthaceae* mediated by immunoglobulin E (IgE) is prevalent in semidesert countries like Saudi Arabia, Iran, and Kuwait. In particular, among Iranian allergic patients, allergy to *A. retroflexus* pollen has been reported with a high positive rate of 68.8% [4, 5]. New ways of treating allergies are based on the use of new adjuvants that induce Th1 responses. The application of probiotic expression systems, which in addition to their adjuvant properties, can transmit allergens to the mucosal surfaces, is one of the exciting new areas in the allergy treatment [6, 7]. The recombinant protein expression in bacterial hosts has been paid increasing attention for both the production and delivery of proteins. Li et al. expressed food-grade *β*-galactosidase through the recombinant *L. lactis* with the potential to alleviate lactose intolerance symptoms [8]. Lee et al. reported the expressing LZ8 protein by *L. lactis* ameliorates nonalcoholic fatty liver and early atherogenesis in cholesterol-fed rabbits [9]. Kalicka et al. investigated the effects of xylitol, erythritol, maltitol, and isomalt on the survival of Bifidobacterium BB-12 during freezing the ice cream over 28 days and reported in all the samples, and bifidobacteria counts remained above 8 log cfu/g [10]. Ayar et al. assessed the viability of *Lactobacillus acidophilus* (ATCC 4357D-5) and *Bifidobacterium animalis* subsp. *lactis* (ATCC 27536) in the ice cream after adding dietary fibre-rich. They said that these products could be used in ice cream with the improved survival of the probiotic bacteria [11].

Lactic acid bacteria (LAB) are a series of Gram-positive bacteria such as Lactococcus, Lactobacillus, Leuconostoc, Pediococcus, Streptococcus, and Oenococcus species. LAB are employed in the production of fermented foods from agricultural raw materials, including milk, cereals, meat, and vegetables. Additionally, LAB strains are the most prevalent microorganisms applied as probiotics. Probiotics defines as alive microorganisms which confer the health effects when administered in adequate amounts [12, 13]. There are several studies on the use of the recombinant probiotic bacteria in the treatment of allergy diseases. Rigaux et al. demonstrated intranasal administration of the genetic modified *L. plantarum* induces of Derp-1 which decreases the development of the Th2 allergic response by a reduction in specific IgE and the induction of allergen-specific IgG2a antibodies [14]. Ai et al. evaluated the effect recombinant LAB on the suppression of dust mite allergy by the mucosal delivery system. Recombinant *L. lactis* (LLDM) was engineered to deliver the Der p2 derivative to the intestinal mucosal surface. Oral immunotherapy of r-*L. lactis* with 2 × 10^9^ cfu/ml dose was done for 5 days per week for 14 days. Oral administration of r-*L. lactis* alleviated the Der p2-induced airway inflammation, as shown by the reduced inflammatory infiltration and a reduction in Th2 cytokines in bronchoalveolar lavage [15].

The objectives of the present study are to investigate the effect of recombinant *L.lactis* in reducing allergic response against *Amaranthus retroflexus* pollens. To investigate the efficacy of the recombinant strain in the treatment of the allergy, the functional ice cream was prepared, and the performance was evaluated through animal model.

## 2. Materials and Methods

### 2.1. Bacterial Strains, Plasmids, and Growth Conditions

*L. lactis* NZ1330 (MobiTec Co, Germany) was considered the host for cloning and expression. pNZ7025 (MobiTec Co, Germany) was applied as the cloning and expression vector for the production of protein. *L. lactis* NZ1330 which was grown in M17 medium at 30°C (Quelab, Canada) supplemented with 1.5% glucose and 200 *μ*g/ml D-alanine without aeration. *Escherichia coli* was cultured on LB agar and broth (Merck, Germany) and incubated at 37°C with a shaking of 180 rpm.

### 2.2. Enzymes and Reagents

Taq DNA polymerase, PFU, Phusion High-Fidelity DNA Polymerase, dNTPs, DNA marker, restriction enzymes, T4 DNA ligase, Plasmid Miniprep Kit, gel extraction kit, and low molecular weight protein marker were supplied from Thermo Fisher Scientific (The USA), NEB (The USA), Roche (Germany), and Parstous (Iran). Mouse interleukin (IL)-4, TGF-*β*, and interferon gamma (IFN-*γ*) enzyme-linked immunosorbent assay (ELISA) kits were obtained from Karmania Parsgene (Iran), and a IgE antibody ELISA kit was obtained from Parstous (Iran), respectively. Phytohaemagglutinin (PHA), culture medium RPMI 1640, fetal bovine serum (FBS), and penicillin/streptomycin were obtained by Gibco (UK).

### 2.3. Gene Identification, Codon Optimization, and Recombinant Expression Vector Construction

**Figure 1:**
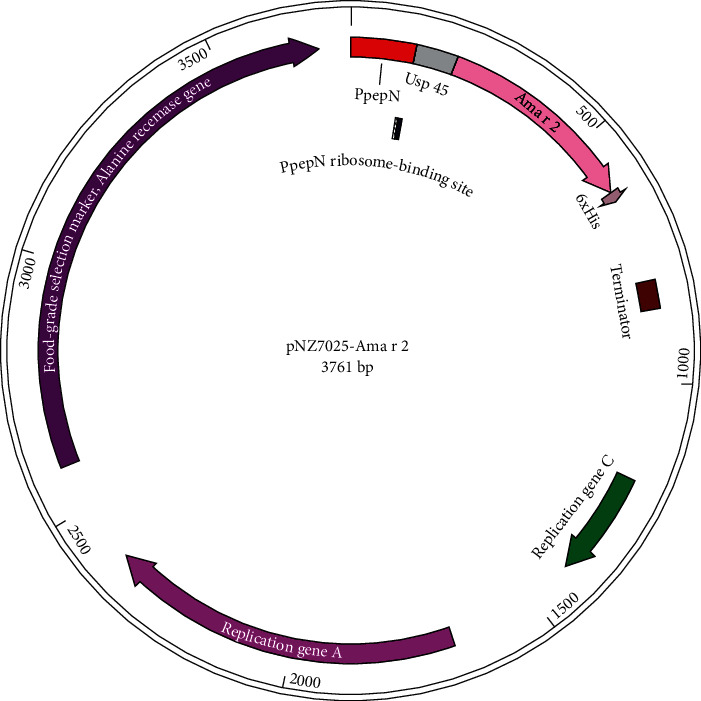
A schematic diagram of the *L. lactis* NZ1330 expression plasmid, pNZ7025-Ama r 2.

There are 133 amino acids in the Ama r 2 sequence. This sequence was provided from the National Center for Biotechnology Information (NCBI) with an accession number of FJ899746.1. The Ama r 2 encoding sequence was codon-optimized for the appropriate expression in *L. lactis* NZ1330. The signal peptide Usp45 (81 bp in length) and the Ama r 2 gene, which carries the restriction sites of *Sph* I and *Spe* I at each terminal, start and stop codon and 6×His tag (Figure 1), were synthesized by Bioneer (South Korea) in the pBHA cloning vector (NCBI accession No. of optimized gene = MT036104).

After the digestion of the pBHA vector by *Sph* I and *Spe* I restriction enzymes, the respective gene was purified and then cloned into a digested pNZ7025 vector. Ligation was carried out for 15 min at ambient temperature using a T4 quick ligation kit. The pNZ7025-Ama r 2 was cloned directly in *L. lactis* through electroporation. Positive colonies can grow on M17 agar without D-alanine, approved by direct colony PCR using pNZ7025-F (AGATCTGTCGACCTGCAGT) and pNZ7025-R (CTATCGAAAGCGAAATCAAACG) primers specifically designed for the pNZ70225 vector. The PCR conditions were as follows: initial denaturation at 94°C for 10 min, 32 cycles (94°C for 30 sec, 56.2°C for 30 sec, and 72°C for 30 sec), followed by a final elongation at 72°C for 10 min. Moreover, the plasmid DNA resulting from the positive clones was purified and introduced to DNA sequencing [16].

### 2.4. Sodium Dodecyl Sulfate Polyacrylamide Gel Electrophoresis and Western Blot Analysis

SDS-PAGE and western blot analyses were done for the samples which cultured during 5 days. Thirty microliters of M17 broth and the supernatant of the sonicated *L. lactis* was mixed with 10 *μ*l of 4X loading. The mixture was boiled at 95°C for 5 min and then electrophoresed on 12% SDS gel electrophoresis. For western blot, after the electrophoresis, the refolded protein was blotted onto polyvinyl difluoride (PVDF) membrane in western blot transfer apparatus (Bio-Rad, USA). The gel was covered with the transfer buffer comprised of Tris (25 mM), glycine (190 mM), and methanol (20%). The transfer was conducted at a constant current of 300 mA in a cold room for 15 min. After washing and blocking with 2% bovine serum albumin (BSA) for 16 h at 4°C, membranes were incubated with the monoclonal anti-polyhistidine peroxidase conjugate antibody (Sigma Aldrich, Saint Louis, USA) at 1 : 5000 dilutions at RT for 3 h. Finally, the blot washed with PBS and subjected to chemiluminescense substrate based on the recommendations of the manufacturer [17].

Blocking was performed with 2% BSA in PBS at 4°C overnight. It was subsequently washed thrice with PBS and probed with.

### 2.5. Site-Directed Mutagenesis

The purpose of SDM was to modify the construct of the vector containing Ama r 2, because the expression of this protein was not observed in SDS-PAGE and western blot. For this purpose, 5 primers were designed whose sequences are presented in Table 1.

The correct design of primer, the application of the polymerase with the lowest error rate, and proper hybridization are the key factors in the appropriate conduct of this technique. PCR was carried out for each of the pair primers of SDM-F1 and SDM-R1 and SDM-F2 and SDM-R2 under the following conditions: initial denaturation at 95°C for 5 min, then 15 cycles (95°C, 30 sec; 60°C (56°C for second pair primers), 30 sec; and 72°C, 2 min) followed by a final elongation at 72°C for 10 min. In the next stage, the hybridization of the replicated strands was performed so that the complementary strands paired with each other. For this purpose, the 10X hybridization buffer was prepared (NaCl 1000 mM, Tris-HCl 100 mM, pH = 7.6, and EDTA 10 mM, pH = 7.6). Nine *μ*l of the PCR product with the first pair primers and 9 *μ*l of that with the second pair primers were mixed with 2 *μ*l of the hybridization buffer under the following temperature cycles: 95°C for 10 min, vortex for 2-3 sec, 80°C for 3 min, 70°C for 3 min, 60°C for 3 min, 45°C for 3 min, and finally, ambient temperature. After hybridization, the excessive salts were removed from the final product so that no perturbation would be created in electroporation. After that, electroporation was conducted based on the previously mentioned protocol. The colonies grown on M17 agar were subjected to direct colony PCR with the specific primers of the vector so as to confirm the presence of the insert-containing vector. However, since some colonies might have belonged to the recombinant bacteria with the primary insert-containing vector, we should have distinguished between the colonies with the primary and modified vectors. To that end, a primer was designed (SDM-F3) whose 3′ end exactly overlapped the 8 nucleotides whose removal was the final goal. Consequently, direct colony PCR was performed on the picked up bacteria using SDM-F3 and SDM-R2 pair primers. In all the cases, in order to observe the bands, agarose gel 1% was prepared in TBE buffer and run at 100 V for 30 min [18].

### 2.6. The Production of Probiotic Ice Cream

For the probiotic ice cream formulation, 57.92% skimmed milk, 20.87% heavy cream (40% fat), 2.71% dry milk, 17% sugar, 0.5% stabilizer and emulsifier (Pulsgard), and 1% vanillin flavor were used. At first, the milk and cream were poured into the formulation tank and heated slowly. When the temperature reached 50°C, the skim milk powder, dry milk, pulsgard, sucrose, and vanillin were added. The ice cream mixture was then pasteurized at 60°C for 30 minutes. Afterwards, the ice cream mixture was cooled to 30-40°C, r-*L. lactis* NZ1330 with 10^12^ CFU/ml added, and incubation was done for 6 h. They kept the mixture in the refrigerator for 24 h to allow the ripening process. It was then added to the ice cream maker and processed for 20 min. To evaluate the viability of the r-*L lactis* NZ1330 strain in the product, before freezing and during storage, every 10 days sampling was performed [19].

### 2.7. The High Production of the Ama r 2 Gene for Sensitization

To produce a large-scale Ama r 2 protein, recombinant *E. coli* containing pET-21b-Ama r 2 constructs was used [4]. The produced rAma r 2 was purified from the soluble phase lysate by an Ni-NTA agarose according to the manufacturer's instructions. The protein concentration was determined according to Bradford's method. The purified protein was then subjected to reducing SDS-PAGE. Proteins are dialyzed (12 kDa cut-off) after purification to remove small molecules such as imidazole from the chromatographic product and changing the buffer conditions [20].

### 2.8. Animals

**Figure 2:**
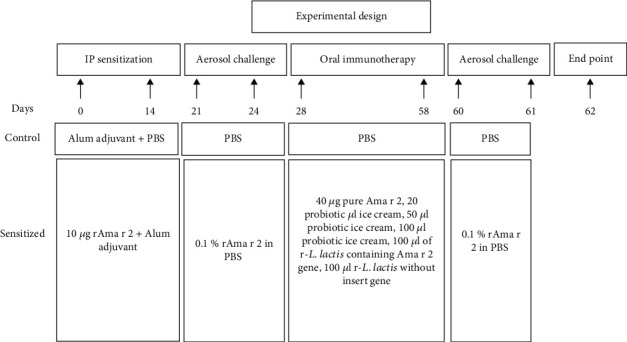
Experimental design. Seven groups of mice (*n* = 5 per group) were used in the experiment. Abbreviations: rAma r 2: recombinant *Amaranthus retroflexus* pollen; ip: intraperitoneal injection.

35 Balb/c mice (male, 6-8 weeks) were purchased from the Pasteur Institute of Iran. Animal keeping procedures were conformed according to the standard guidelines of animal care and were approved by the Animal Ethics Committee of Ferdowsi University of Mashhad (IR.UM.REC.1398.066). After one week of habituation, they were randomly divided into 7 groups (Figure 2).

### 2.9. Sensitization of Mice

Mice sensitization was performed according to a standard protocol with two intraperitoneal injections containing 10 *μ*g r-Ama r 2 plus alum adjuvant (2 mg) on days 0 and 14, and then subsequently, they were sensitized by the inhalation using the r-Ama r 2 solution at a concentration of 0.1 mg/ml and using a nebulizer since day 21, each day for 20 minutes over four consecutive days [21].

### 2.10. Oral Immunotherapy and Evaluation of Th1, Th2, and Treg Response Indices

Treatment was performed orally with gavage needles, 4-5 times a week for a month at specified amount and concentrations. The mice were again exposed to the rAma r 2 allergen solution on days 60 and 61 using a nebulizer. At day 62, mice were euthanized, and serum and spleen of different groups were separated for the evaluation of the humoral and cellular immune responses. Serum from different groups was collected to evaluate changes in the IgE antibody levels and analyzed using the ELISA technique. The spleen cells were removed by sterile forceps and scissors and transferred to a 1.5 ml microtube from an incomplete cold RPMI medium. The cells were then ejected into petri dishes by injecting 2-3 ml of incomplete RPMI with a syringe needle from one side of the spleen. Next, the cells were collected and poured into a sterile 15 ml falcon (all these steps should be performed on ice to prevent cell death). The tubes were then centrifuged at 1500 rpm for 4 min at 4°C, and the supernatant was removed. After that, the cells attached to the falcon body were removed and suspended. For the RBC lysis, 5 ml of cold lysis buffer or ammonium chloride buffer was added to the suspension and gently mixed by inverting the tube. The tubes were incubated on ice for 5 min and washed twice with 5 ml of sterile PBS to remove ammonium chloride. Next, 2 ml of complete RPMI was added to the cell suspension. Then, 20 *μ*l of cell suspension was merged with 20 *μ*l of trypan blue dye, and cells were counted with a neobar slide under the microscope. Cells were cultured in 24-well flat-bottomed culture plates in duplicate at a density of 2 × 10^6^ cells/ml and stimulated with Ama r 2 (10 *μ*g/ml), PHA (1 *μ*g/ml), or media at 37°C. The cell supernatant was collected after 72 h to measure TGF-*β*, IFN-*γ*, and IL-4 cytokines [22].

### 2.11. Statistical Analysis

Descriptive statistics, mean, standard deviation, tables, and graphs were used to describe the data. The statistical analyses were performed using GraphPad Prism 8.0.2. The data have been expressed as mean ± standard deviation. Means were compared using one-way analysis of variance (ANOVA) followed by Tukey–Kramer test. All *p* values <0.05 were considered significant.

## 3. Results and Discussion

### 3.1. The Cloning and Transformation of the Expression Vector into *L. lactis* NZ1330

**Figure 3:**
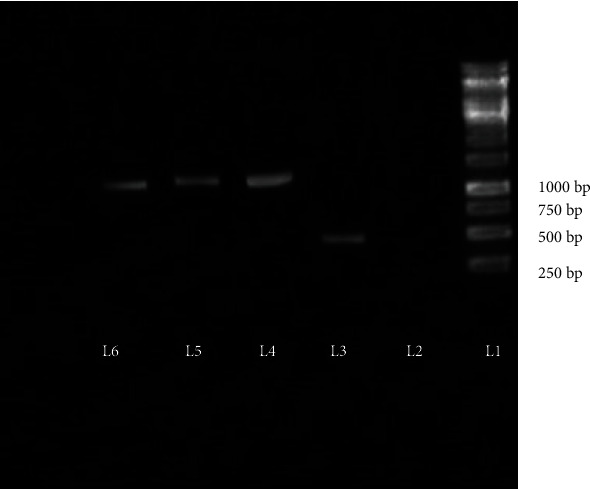
Agarose gel electrophoresis of Ama r 2 PCR products. Lane 1 is a 1 kbp Ladder (Fermentase), lane 2 is a negative control, lane 3 is self-plasmid, and lanes 4, 5, and 6 are the products of the direct-colony PCR of the recombinant bacteria.

Ama r 2 comprises an open reading frame of 399 bp which encodes 133 amino acids with a calculated pI of 4.46 and a molecular mass of 14.2 kDa. The presence of the Ama r 2 gene in *L. lactis* was examined with direct-colony PCR. Plasmid primers amplified a 1000 bp fragment which contained the Ama r 2 sequence. While PCR was being performed on self-plasmid colony, the segment which should have been observed on the agarose gel was 488 bp in length (Figure 3).

Both the medium and the lysed bacteria were analyzed using SDS-PAGE and western blot. They were also tested with different methods of protein sedimentation, including ammonium sulfate precipitation and purified by nickel column, but the 14.2 kDa band was not observed.

### 3.2. The Modification of pNZ7025-Ama r 2 Construct with Site-Directed Mutagenesis

The spacing between the RBS and the translation initiation codon remarkably differs in naturally occurring mRNAs with a mean length of 7–8 nt. Extremely long or short spacing between the RBS and the start codon could be harmful to the effective translation initiation [23]. Consequently, it was decided to decrease this spacing from 13 nucleotides to 8 ones to assess its effect on the expression of Ama r 2. SDM is a reliable and precise technique in decreasing or increasing the number of nucleotides based on the predicted purpose for the genomic construct. The key point is the prevention of the accidental mutation during PCR. Therefore, the enzymes with low mutation rates were used, and the number of cycles were reduced to 15 [24]. Forward and reverse primers are centered on the suitable base change(s) and perfectly overlap in both directions. The primer extension surrounding the entire plasmid creates a copy of the template with the interest mutation. The primers should have a length of 25–45 nucleotides leaving a minimum of 10–15 nucleotides with a perfect matching sequence at both ends. In order to replicate the two strands and remove the excessive nucleotides, the primers were designed such that one of them overhung in the mutation location and the other one replicated the region after which it should have been removed. The next stage was the hybridization of the two open ends of the replicated strands [25]. The mutated vector is transferred to *L. lactis* NZ1330 competent cells via electroporation based on the protocol mentioned earlier. In fact, the two open ends of the plasmid strand adhere to each other using the bacterium ligase, and a new plasmid with a circular construct is formed. This process is called the ligation independent cloning. The grown colonies were evaluated on M17 agar to find out whether the recombinant vector was modified. In order to distinguish between the bacteria containing the mother vector and those with the modified one, a primer (SDM-F3) was designed whose 3′ end exactly matched the 8 nucleotides which were going to be removed from the vector construct. If the vector were not modified, the primer would match the segment, be replicated, and its band would be observed on the agarose gel. On the other hand, if the 8 nucleotides were removed, the primer could not attach with the segment and consequently, no band would be observed on the gel.

**Figure 4:**
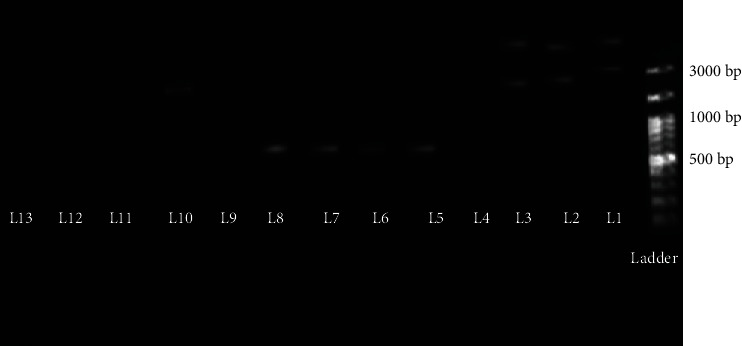
PCR products of 3 colonies with modified vectors growth on M17 agar. Lane No.: ladder 100 bp plus 3 kb (DM2300), lanes 1-3 contributed to the vectors which extracted, lane 4: negative control of PCR with specific primers (pNZ7025-F and pNZ7025-R), lane 5: positive control for PCR with specific vector primers. Lanes 6-8 contributed to PCR products of extracted vector with specific pNZ7025 primers, lane 9 is a positive control for PCR with SDM-F3 and SDM-R2, lane 10 is a negative control for PCR with SDM-F3 and SDM-R2, and lanes 11-13 are PCR products of 3 modified vector with SDM-F3 and SDM-R2.

As a result, the grown colonies on the medium were analyzed using two pair primers: firstly, with the specific pair primers of the vector to ensure the presence of the pNZ7025 vector and secondly, the bacteria with positive responses were analyzed using SDM-F3 and SDM-R2. As can be seen in Figure 4, the results of the agarose gel of the PCR products of the three picked up colonies demonstrated that all the three bacteria contained the recombinant vector. In addition, all the three vectors were free of the 8 nucleotides (The result of the PCR with SDM-F3 and SDM-R2 was negative). Moreover, the result sequencing indicated that these changes were successfully made.

### 3.3. SDS-PAGE and Western Blotting

**Figure 5:**
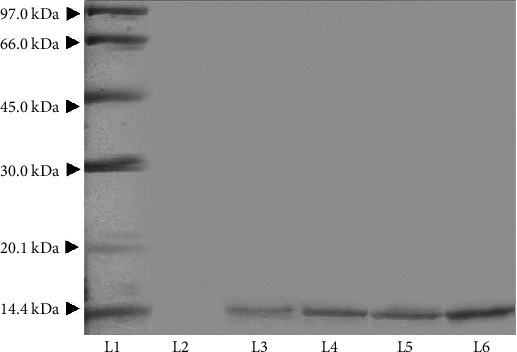
Western blot analysis using monoclonal anti-polyhistidine-peroxidase antibody as a direct antibody. Lane 1: low molecular weight protein ladder, and lanes 2-6 are *Lactococcus lactis* cell lysate from the bacterial growth from day 1 to 5.

Western blotting is a widely used analytical technique in cell and molecular biology to detect specific proteins in a mixture of proteins [26]. As the detection and separation of recombinant proteins are usually a laborious process, epitope tags are very versatile tools which are normally utilized to resolve this issue. Here, the extra sequences of histidine are regularly incorporated into the C-terminals of the target protein. These sequences of amino acids are able to represent epitopes for certain binding partners like antibodies [27]. The bacteria whose vectors had been modified through SDM were cultured on M17 broth+glucose for 5 days. The cells were lysed using a sonicator, and the cellular extract was collected via centrifugation and subsequently underwent SDS-PAGE. The bands were then transferred to the PVDF membrane, blocked, and the membrane reacted with the anti-histag antibody. The results of western blot are illustrated in Figure 5. As the results show, all the bands are about 14.2 kDa compared with the protein ladder, revealing that the bands belonged to Ama r 2. The protein expression production was assessed by image analysis of the western blotting scans using the ImageJ software. The largest amount of expression was done on the fifth day, which was estimated to be 10 *μ*g/ml after the band analysis in comparison to the standard sample.

The application of genetically modified and/or recombinant organisms causes many concerns. In order to lower these concerns, expression systems—also known as food grade—have been introduced (de Castro et al. 2018). The distinguishing characteristic of such vectors is the lack of antibiotic resistance markers (Tagliavia and Nicosia 2019). Numerous studies have been conducted concerning the application of LAB hosts in the expression of allergens. Nasiraie et al. cloned Che a 2 allergen, as a candidate for oral immunotherapy, into *L. lactis* which selected marker was erythromycin, and the expression induction was done with 2% lactose (Nasiraie et al. 2013). Glenting et al. expressed Ara h 2, the main peanut allergen, in *L. lactis*. The Ara h 2 gene was cloned into pAMJ399 plasmid containing the P170 promoter and the SP310mut2 signal sequence. Also, the recombinant bacteria were selected based on the resistance to erythromycin (Glenting et al. 2007). In the studies carried out on the production of recombinant allergens in prokaryotic and even probiotic hosts, in order to select the recombinant strain in the culture medium, the resistance marker to antibiotics such as erythromycin and chloramphenicol is used as a selector so that the vector-receiving strain can survive in the medium containing antibiotic (Rosano and Ceccarelli 2014). Based on our current knowledge, the utilization of food grade vectors and the hosts fulfilling food industry standards for allergen expression either have not been performed. FDA suggests not employing antibiotics for the selection of bacteria. In particular, beta-lactam antibiotics, like penicillin, should not be applied during the production of a therapeutic product for human consumption; this may allow the transfer of the resistance gene to the intestine microflora, which can cause problems in curing infectious diseases (Bachman 2013; Food and Administration 2015). As a result, in a previous research, Vasiee et al. investigated the resistance of the r-*L. lactis* NZ1330 containing the pNZ7025-Ama r 2 plasmid against 9 antibiotics, namely, vancomycin (30 *μ*g), fosfomycin (200 *μ*g), kanamycin (30 *μ*g), gentamycin (10 *μ*g), neomycin (30 *μ*g), cefixime (5 *μ*g), ciprofloxacin (5 *μ*g), ampicillin (10 *μ*g), and erythromycin (15 *μ*g). Their results revealed that this recombinant strain did not resist against any of these antibiotics (Vasiee et al. 2019).

### 3.4. The Production of Probiotic Ice Cream and Counting Viability of *L. lactis* NZ1330

**Figure 6:**
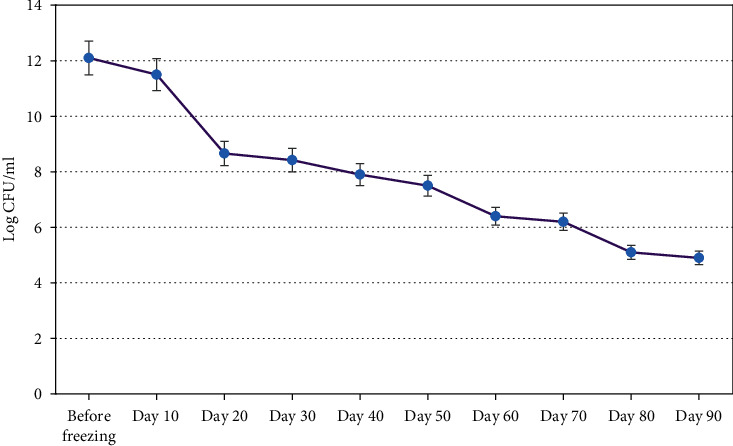
Bacterial viability results for r-*L. lactis* NZ1330 in ice cream produced within 90 days.

Ice cream containing r-*L. lactis* NZ1330 was prepared for oral use for treating sensitized mice to *Amaranthus retroflexus* pollen. In this study, probiotic bacteria were counted within 90 days of production (every 10 days) with the serial dilution method on M17 agar medium (Figure 6). The number of bacteria started at 12 _log_ CFU/ml on day zero and decreased to about 5_log_ CFU/ml at the end of the third month. The highest decrease was observed in the first month, which was about 4 cycles. The viability of probiotic bacteria would be affected during the production, storage, and consumption of the probiotic ice cream. Cold shock, lack of sufficient amount of essential amino acids in ice cream, and overrun during ice cream production are considered as deadly factors [28].

### 3.5. Serum IgE Levels after Oral Immunotherapy

**Figure 7:**
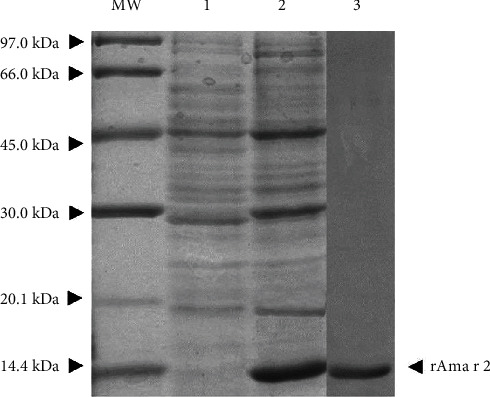
SDS-PAGE analysis of rAma r 2, after expression, after induction, and after purification. Notes: MW: molecular mass markers; 1: *E.coli* precipitate lysed without IPTG induction; 2: *E. coli* lysis precipitated after induction by IPTG; and 3: Ni-purified rAma r 2.

Allergen sensitization with rAma r 2 in alum adjuvant resulted in the elevated serum IgE antibody level in all mice of the target group. To sensitized the mice, high amount of rAma r 2 was required which produced by the pET-21b in *E. coli* host (Figure 7).

**Figure 8:**
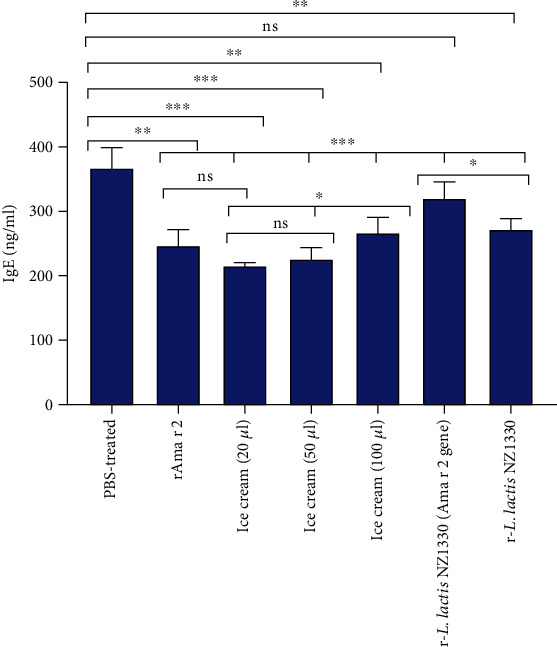
Total serum IgE levels after immunotherapy. ns: not significant; ^∗∗∗^*p* < 0.001, ^∗∗^*p* < 0.01. and ^∗^*p* < 0.05 versus the normal or PBS-treated group. Normal group: untreated and unsensitized group (PBS and alum-sensitive and treated with PBS), PBS-treated group: sensitized with rAma r 2 and PBS-treated, rAma r 2 group: sensitized with rAma r 2 and treated with pure rAma r 2 at a concentration of 40 *μ*g/ml, ice cream (20 *μ*l): sensitized with rAma r 2 and treated with 20 *μ*l ice cream containing r-*L. lactis* NZ1330, ice cream (50 *μ*l): sensitized with rAma r 2 and treated with 50 *μ*l of ice cream containing probiotic bacteria, ice cream (100 *μ*l): sensitized with rAma r 2 and treated with 100 *μ*l of ice cream containing r-*L. lactis* NZ1330, r-*L. lactis* NZ1330 (Ama r 2 gene): sensitisized to rAma r 2 and treated with probiotic bacteria r-*L. lactis* NZ1330 which contains the Ama r 2, and r-*L lactis* NZ1330: sensitized with rAma r 2 and treated with probiotic bacteria lacking the Ama r 2 gene.

**Figure 9:**
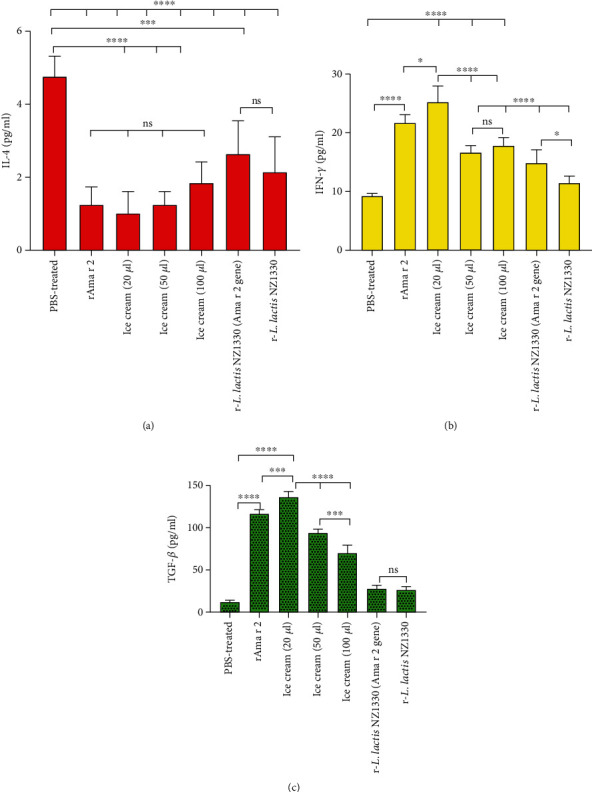
Cytokine levels IL-4 (a), IFN-*γ* (b), and TGF-*β* (c) in supernatant of spleen cell culture after the restimulation with rAma r 2. ns: not significant; ^∗∗∗∗^*p* < 0.0001, ^∗∗∗^*p* < 0.001, ^∗∗^*p* < 0.01, and ^∗^*p* < 0.05 versus the normal or PBS-treated group. Normal group: untreated and unsensitized group (PBS and alum-sensitive and treated with PBS), PBS-treated group: sensitized with Ama r 2 and PBS-treated, rAma r 2 group: sensitized with Ama r 2 and treated with pure rAma r 2 at a concentration of 40 *μ*g/ml, ice cream (20 *μ*l): sensitized with rAma r 2 and treated with 20 *μ*l ice cream containing r-*L. lactis* NZ1330, ice cream (50 *μ*l): sensitized with rAma r 2 and treated with 50 *μ*l of ice-cream containing probiotic bacteria, ice cream (100 *μ*l): sensitized with rAma r 2 and treated with 100 *μ*l of ice cream containing r-*L. lactis* NZ1330, r-*L. lactis* NZ1330 (Ama r 2 gene): sensitisize with Ama r 2 and treated with probiotic bacteria r-*L. lactis* NZ1330 which contains the Ama r 2, and r-*L lactis* NZ1330: sensitisized with Ama r 2 and treated with probiotic bacteria lacking the Ama r 2 gene.

Although there are various methods for measuring this immunoglobulin, the most important and precise method is ELISA [29]. However, the IgE-specific antibody production was not observed in control (untreated) mice whose sensitization process was performed by intraperitoneal injection of alum adjuvant in PBS. As shown in Figure 8, the effect of all treatment groups except suspended r-*L. lactis* (contain insert gene) on the reduction of IgE was significantly different compared to the control group. The treatment group with a daily consumption of 20 and 50 *μ*l of probiotic ice cream containing r-*L. lactis* NZ1330 showed a significant difference (*p* < 0.001) compared to the PBS-treated group. There was also a significant difference between the treatment group with 100 *μ*l probiotic ice cream (*p* < 0.01) with the control group. There was no significant difference between the treatment groups with a daily consumption of 20 and 50 *μ*l of probiotic ice cream, although there was a significant difference at the *p* < 0.05 level with the 3 different ice cream formulations. One of the long standing immunotherapy methods is using low doses of pure allergen for treating allergy which in this study, recombinant allergen produced, purified, and dialyzed at a concentration of 40 *μ*g/ml and was used to evaluate the efficacy of this treatment. As shown in Figure 9, the pure allergen treatment group did not show a significant difference compared to the 20 *μ*l daily functional ice cream intake group, although the difference was significant at the *p* < 0.01 level compared to the PBS-treated group. To ensure the efficacy of the recombinant probiotic strain for the treatment of allergic rhinitis, the recombinant bacterium with the Ama r 2 gene as well as the recombinant bacterium with the pNZ7025 vector without the Ama r 2 gene that were diluted to the 10^12^ CFU/ml in PBS buffer was also included in treating groups. Therapeutic effect of the r-*L.lactis* NZ1330 group (Ama r 2 gene) was not significantly decreased IgE compared to the PBS treatment group. However, the group treated with recombinant probiotic bacteria (without Ama r 2 gene) had a significant difference at the *p* < 0.01 level with the group treated with PBS. r-*L. lactis* NZ1330 (Ama r 2 gene) treatment groups and r-*L. lactis* NZ1330 had a significant difference at the *p* < 0.05 level.

### 3.6. The Evaluation of the Spleen Cell Cytokine Production after Oral Immunotherapy

#### 3.6.1. Il-4

IL-4 is the major cytokine in stimulating B lymphocytes to produce IgE. This cytokine also prevents antibody class switching to IgG2a and IgG3. IL-4 inhibits macrophage activity and many of the positive effects of interferon-gamma to increase the production of cytokines such as IL-1 [30]. The IL-4 concentration in supernatant of spleen cells stimulated with rAma r 2 is shown in Figure 9(a). The secretion of this cytokine decreased after immunotherapy in compared to the PBS group, which was significantly different in all treatment groups. Between rAma r 2 and ice cream groups with 20, 50, and 100 *μ*l of treatment and between the pure r-*L. lactis* NZ1330 group (Ama r 2 gene) and pure r-*L. lactis* NZ1330 showed no significant difference. There was a significant difference between the effect of probiotic ice cream groups compare to the PBS group at *p* < 0.0001. Difference between r-*L. lactis* NZ1330 groups (Ama r 2 gene) and pure r-*L. lactis* NZ1330 and PBS-treated group was also significant at the *p* < 0.001 level.

#### 3.6.2. IFN-*γ*

IFN-*γ* is a cytokine that is usually produced by Th0 and Th1 lymphocytes as well as most CD8+cells. The transfection of the IFN-*γ* gene begins immediately after the stimulation of the cell with the antigen, and IL-2 and IL-12 exert a resonant effect on it. This cytokine plays a major role in the development of cellular immune-mediated diseases. This cytokine is the most important activator of macrophages and unlike IL-4, IFN-*γ* decreases IgE levels. It can also modulate the antibody class to IgG2a and IgG3 subunits by affecting B cells and prevent the IgG1 and IgE production [31]. The result of the evaluation of IFN-*γ* levels after oral immunotherapy is shown in Figure 9(b). All treatment groups showed higher IFN-*γ* production than the PBS treatment group. This increase was significant in the treatment group with 40 *μ*g/ml allergen compared to the PBS group at *p* < 0.0001. The difference between the 40 *μ*g/ml allergen and 20 *μ*l functional ice cream was significant at *p* < 0.05. Also, the difference between the three probiotic ice cream groups were significant at *p* < 0.0001. There was no significant difference between the treatment group of 50 and 100 *μ*l of probiotic ice cream. There was a significant difference between the consumption of bacteria with the Ama r 2 gene and no Ama r 2 gene at the *p* < 0.05 level. Also, there was a significant difference between ice cream groups with 50 and 100 *μ*l daily consumption and pure bacteria with and without the Ama r 2 gene at the *p* < 0.0001 level.

#### 3.6.3. TGF-*β*

TGF-*β* is an anti-inflammatory cytokine that is produced by a large number of immune cells, including regulatory T lymphocytes and macrophages. This cytokine has many anti-inflammatory properties and is present on a large number of immune cells containing the receptor and can alter the type of antibody to IgA, which is of particular importance in mucosal immune responses [32]. Based on ELISA results after immunotherapy, the TGF-*β* cytokine production was significantly increased in the 6 treatment groups compared to the normal group (Figure 9(c)). There was a significant difference between the PBS-treated and the all other treated groups. Significant differences were observed between the allergen treatment group and 20 *μ*l of functional ice cream at the *p* < 0.001 level. There was also a significant difference between the three ice cream groups at *p* < 0.0001. However, there was no significant difference between the treatment groups with pure probiotic bacteria containing the Ama r 2 gene and those without the pure gene. Also, there was a significant difference between the treatment groups of 50 and 100 *μ*l of probiotic ice cream per day (*p* < 0.001).

The current study explored the potential efficacy and mechanism of action of r-*L. lactis* NZ1330 against the development of aspiration allergy manifestations. The r-*L. lactis* contains the Ama r 2 gene as a means of producing and transmitting this allergen (simultaneously) to intestinal epithelial cells. Because of the patient's preference for the drug to be used until the injection, the efficacy of oral immunotherapy was chosen to treat the sensitive mice. To investigate the effect of bacterial counts on allergy treatment, three ice cream volumes were used (20, 50, and 100 *μ*l ice cream containing 10^12^ CFU/ml r-*L. lactis* NZ1330). The results showed that by increasing the volume of ice cream gavage, the evaluated parameters became weaker. The results of using 20 *μ*l of ice cream daily had a better response than 50 and 100 *μ*l groups. Regarding the evaluation of cytokines, after ice cream with a volume of 20 *μ*l, the pure allergen group provided the best response, but for IgE, the ice cream group with a volume of 50 *μ*l was second in the reduction of this antibody. In the case of IL-4, the 50 *μ*l ice cream group showed a better response to ELISA compared to the 100 *μ*l group, whereas the IFN-*γ* treatment group received 100 *μ*l probiotic ice cream. In addition, analysis TGF-*β* showed that the treatment group containing 20 *μ*l of probiotic ice cream showed a better response to pure allergen. Hajavi et al. reported that sublingual immunotherapy by 12.5 *μ*g/dose of rChe a 3 encapsulated in PLGA was effective than other doses (25 and 50 *μ*g) in reducing the eosinophilic infiltration and Th2 response [33]. Charng et al. showed that some recombinant LAB containing Der p 5 allergen when orally treated are able to inhibit the allergen-induced airway allergic inflammation [34]. Rigaux et al. demonstrated the intranasal administration of the genetic modified *L. plantarum* induces of Derp-1 which decreases the development of the Th2 allergic response by a reduction in specific IgE and the induction of allergen specific IgG2a antibodies [14]. Ai et al. evaluated the effect of the recombinant LAB on the suppression of dust mite allergy by the mucosal delivery system. Recombinant *L. lactis* (LLDM) was engineered to deliver the Der p2 derivative to the intestinal mucosal surface. Oral immunotherapy of r-*L. lactis* with 2 × 10^9^ cfu/ml dose was done for 5 days per week for 14 days. Oral administration of r-*L. lactis* alleviated the Der p2-induced airway inflammation, as shown by the reduced inflammatory infiltration and a reduction in Th2 cytokines in bronchoalveolar lavage [15].

## 4. Conclusion

The food-grade production of recombinant proteins in Gram-positive bacteria, especially in *Lactococcus lactis*, is one of great interest in the areas of recombinant enzyme production, industrial food fermentation, gene, and metabolic engineering, as well as antigen delivery for oral vaccination. In this study, the expression of the recombinant allergen, Ama r 2, was evaluated in the probiotic host, *L. lactis* NZ1330. The recombinant bacteria were fed to mice, and their effects on allergy treatment were evaluated. IgE, IL-4, TGF-*β*, and IFN-*γ* were evaluated using ELISA. The best response was seen in the treatment group with 20 *μ*l of ice cream daily. Generally, the food grade *L. lactis* NZ1330 can be considered as a good vehicle to deliver therapeutic proteins to target sites in the body.

## Figures and Tables

**Table 1:** The primers used in site-directed mutagenesis.

Name	Sequence (5′ to 3′)
SDM-F1	GGAGAAGGCATGAAAAAGAAAATTATTTCAGCTATTTTAATGTC
SDM-R1	TAAATATTCAGTATTAATATAATTATATCAAATTGAATAG
SDM-F2	AAAATTATTTCAGCTATTTTAATGTCTAC
SDM-R2	CTTTTTCATGCCTTCTCCTAAATATTCAGT
SDM-F3	AGGAGAAGGCATGCATGTATG

## Data Availability

All data generated or analyzed during this study are included in this published article.
